# Non-pharmaceutical interventions to combat COVID-19 in the Americas described through daily sub-national data

**DOI:** 10.1038/s41597-023-02638-6

**Published:** 2023-10-21

**Authors:** Michael Touchton, Felicia Marie Knaul, Héctor Arreola-Ornelas, Thalia Porteny, Óscar Méndez Carniado, Marco Faganello, Calla Hummel, Silvia Otero, Jorge Insua, Fausto Patino, Eduardo Undurraga, Pedro Pérez-Cruz, Mariano Sanchez-Talanquer, V. Ximena Velasco Guachalla, Jami Nelson-Nuñez, Carew Boulding, Renzo Calderon-Anyosa, Patricia J Garcia, Valentina Vargas Enciso

**Affiliations:** 1 Department of Political Science, School of Arts and Sciences, University of Miami, Miami, Florida USA; 2 Institute for Advanced Study of the Americas, University of Miami, Miami, Florida USA; 3 Leonard M. Miller School of Medicine, University of Miami, Miami, Florida USA; 4 Tómatelo a Pecho, A.C., Mexico City, Mexico; 5 Fundación Mexicana para la Salud, A.C., Mexico City, Mexico; 6 Institute for Obesity Research, Tecnológico de Monterrey, Monterrey, Mexico; 7 School of Government and Public Transformation, Tecnológico de Monterrey, Mexico City, Mexico; 8 Department of Health Policy and Management, Columbia Mailman School of Public Health, New York, New York USA; 9 MAF dataScience, Universidade Estadual de Campinas, Campinas, Brazil; 10 Facultad de Estudios Internacionales, Políticos y Urbanos, Universidad del Rosario, Bogotá, Colombia; 11 Health Policy and Management, School of Biomedical Sciences, School of Government, School of Health Care Management, Universidad Austral, Buenos Aires, Argentina; 12 School of Public Health, University of Buenos Aires, Buenos Aires, Argentina; 13 Universidad Andina, Simón Bolívar, Quito, Ecuador; 14 Escuela de Gobierno, Pontificia Universidad Católica de Chile, Santiago, Chile; 15 Departamento Medicina Interna, Facultad de Medicina, Pontificia Universidad Católica de Chile, Santiago, Chile; 16 Millennium Nucleus for the Study of the Life Course and Vulnerability, Santiago, Chile; 17 Department of Political Science, Colegio de Mexico, Mexico City, Mexico; 18 Department of Government, University of Essex, Essex, England; 19 Department of Political Science, University of New Mexico, Albuquerque, New Mexico USA; 20 Department of Political Science, University of Colorado, Boulder, Colorado USA; 21 Department of Epidemiology, Biostatistics and Occupational Health, McGill University, Montreal, Quebec Canada; 22 Universidad Peruana Cayetano Heredia, Lima, San Martin de Porres Peru

**Keywords:** Research data, Interdisciplinary studies, Policy, Infectious diseases, Public health

## Abstract

This dataset covers national and subnational non-pharmaceutical interventions (NPI) to combat the COVID-19 pandemic in the Americas. Prior to the development of a vaccine, NPI were governments’ primary tools to mitigate the spread of COVID-19. Variation in subnational responses to COVID-19 is high and is salient for health outcomes. This dataset captures governments’ dynamic, varied NPI to combat COVID-19 for 80% of Latin America’s population from each country’s first case through December 2021. These daily data encompass all national and subnational units in Argentina, Bolivia, Brazil, Chile, Colombia, Ecuador, Mexico, and Peru. The dataset includes individual and aggregate indices of nine NPI: school closures, work suspensions, public event cancellations, public transport suspensions, information campaigns, local travel restrictions, international travel controls, stay-at-home orders, and restrictions on the size of gatherings. We also collected data on mask mandates as a separate indicator. Local country-teams drew from multiple data sources, resulting in high-quality, reliable data. The dataset thus allows for consistent, meaningful comparisons of NPI within and across countries during the pandemic.

## Background & Summary

Latin America is one of the regions most affected by the COVID-19 pandemic. Only 8% of the global population lives in Latin America, but the region accumulated 30% of total COVID-19 deaths through August 2022^1^. There is significant variation in the distribution of cases and deaths in each country, but very few subnational or national governments used NPI effectively to combat COVID-19^1–4^.

Data on subnational NPIs are crucial for explaining pandemic outcomes and for building knowledge on how to improve performance in future pandemics^5–15^. These data are relevant beyond pandemics, too, as federal systems of government rely on subnational units to respond to natural disasters and other crises as well as to deliver critical services under normal conditions. Similarly, the process of decentralization has allowed subnational units of unitary countries to implement their own policies, which often diverge from those of the national government. The timeliness, mix, and rigor of national and subnational NPIs in Latin America in our dataset is therefore useful for scholars and practitioners around the world, now and in the future^16^.

The timing, combination, and types of NPI in Latin America varied across and within the countries included in our data since the first COVID-19 case was recorded on February 25 2020, in São Paulo, Brazil^17,18^. Many countries had implemented at least some national restrictions by the end of March 2020, but NPI stringency and type shifted in dramatic waves over the first year of the pandemic and continues to do so in the face of outbreaks^19^. For example, Brazilian and Mexican national governments deferred NPI responsibility to state governments, leading to large-scale variation within each country with no central, evidence-based planning^20^. Understanding this variation is important, since the countries accounted for 17% of the world’s confirmed COVID-19 deaths by March 2021. It is also important for drawing lessons that will inform policy in future regional and global pandemics.

Throughout 2020 and 2021, national leaders relaxed or removed sub-national NPI to balance concerns for COVID-19 transmission with economic imperatives, and declines in mental health due to lockdowns. NPIs were also discontinued due to political, libertarian, and human rights-based controversies around their design and sometimes uneven implementation.

In Latin America, many countries also faced difficulties in collecting sufficient evidence to inform subnational policymaking. This struggle created a patchwork of NPIs within and across countries, which only rarely responded to local variation in COVID-19 cases and deaths because of minimal testing and contact tracing.

Our dataset records the timing, mix, rigor, and type of NPIs adopted at the state, province, department, and regional levels for Argentina, Bolivia, Brazil, Chile, Colombia, Ecuador, Mexico, and Peru^16^. The data covers 80% of Latin America’s population from the first case in each country through December 2021^16,21^, almost two years after the first cases in each country. The data also covers different types of governance^16^, on a gradient of federal and unitary systems, decentralized and centralized, Left, Right, and populist governments at national and subnational levels. The daily data included in the dataset thus fill a gap in subnational, daily data with variables and methods absent from other datasets^16,22^.

Our dataset is distinct from others that record similar data in several ways^16^. First, our coding methods used large teams of native-speaking, in-country researchers to record data, rather than bots or other automated data collection^16^. Next our data is daily by subnational unit, not weekly or monthly, which offers unusual granularity, from the first case in each country through the end of 2021 for all eight countries and all subnational units, covering the first 22 months of the pandemic^16^. This timeframe is longer than other datasets on NPI to combat COVID-19. The total NPI included, the specific indicators for each, and the construction of our index also set our dataset apart from others^16^. In sum, our dataset is unique in its granularity, longitudinal coverage, and use of in-country research teams to code subnational data^16^. Our 53, 411 observations are larger than any other source for the countries in question^16^.

## Methods

As part of the Observatory for the Containment of COVID-19 in the Americas^21^, we collected data on NPIs in each of the eight countries’ subnational territories, beginning with the first reported case in each country^16^. We focus on the state, department, or provincial level of government administration. We present data from February 25, when the first Latin American COVID-19 case was confirmed in São Paulo, Brazil, to the end of 2021, spanning the first 22 months of the pandemic in the region and the first two years for Brazil and Mexico, the region’s two largest countries^16^.

These data include school closures, work suspensions, public event cancellations, public transport suspensions, information campaigns, travel restrictions within states, international travel controls, stay-at-home orders, and restrictions on the size of gatherings^16^. We also collect and report data on mask mandates separately^16^. A literature review at the beginning of the pandemic guided us in the selection of these NPI, which previous scholarship identified as relevant for influencing COVID-19 cases and deaths^23–28^. We also relied on the Oxford COVID-19 Government Response Tracker (OxCGRT) 5.0^29^, which recorded data on national-level policy to identify the 10 most important NPIs. Parallel data collection on NPIs to combat COVID-19 tend to include a subset of our variables^30–33^.

We assembled country teams of local doctors, professors, policy experts, researchers, and university students to examine which policies were in effect, when they were implemented, and whether they remained in effect each day, from the first case detected in the country. We then coded the measure’s policy implementation intensity as partial or total if it was in effect. Tables 1, 2 describe the 10 NPI indicators, their coding, and their values. We assigned the indicators several discrete levels to create possibilities for granular analyses. Scores range from 0 to 1 in discrete levels.

**Table 1:** NPI Variables and Coding.

Variable Name	Description	Coding
Stay at Home	Record orders to “shelter-in-place”	0 - No measures 0.33 - Recommend not leaving the house 0.66 - Partial requirements (specified groups or times) 1 - Require not leaving the house with minimal exceptions (e.g., allowed to leave only once a week, or only one person can leave at a time, etc.)
School Closure	Closure of in-person classes	0 - No measures 0.5 - Recommend closing 1 - Require closing all levels
Workplace Closure	Workplaces closures, restrict working hours, and/or closure of specific economic activities/sectors	0 - No measures 0.5 Partial (or work from home) for some activities, sectors or categories of workers, and times 1 - Require closing
Public Events Cancelled	Prohibition of events, social, cultural, or religious activities and sports	0 - No measures 0.5 - Recommend cancelling 1 - Require cancelling
Restrictions on gatherings	Restrictions on crowds of people	0 - No restrictions 0.33 - Restrictions on very large gatherings (limit is above 1000) 0.66 - Restrictions on gatherings between 11–100 people 1 - Restrictions on gatherings of 10 people or less.

Some data in this table have been published previously in Knaul, F.M. *et al*. Strengthening health systems to face pandemics: subnational policy responses to COVID-19 in Latin America. *Health Affairs*. **41**, 454–462 (2022)^31^.

**Table 2:** Additional NPI Variables and Coding.

Variable Name	Description	Coding
**Variable Name**	**Description**	**Coding**
Public transit suspended	Traffic restrictions, schedules, types, or routes of travel in each state/department	0 - No measures 0.33 - Recommended closing 0.66 - Significantly reduce volume/route/means of transport available 1 - Require closing (or prohibit most citizens from using it)
Information campaign	Measures to disseminate information on health, contagions, prevention measures, tests, and laws	0 - No COVID-19 public information campaign 0.5 - Public officials urging caution about covid-19 1 - Coordinated public information campaign (e.g., across traditional and social media)
Mask mandate	Use of masks and face coverings	0 - No measure 0.33 - Recommended 0.66 - Partial and mandatory 1 - Total/mandatory implementation
Internal travel control	Traffic restrictions, schedules, types, or routes of travel within each state/department	0 - No measures 0.33 - Recommend not to travel between regions/cities 0.66 - Relaxed moving restrictions 1 - Internal moving restrictions in place
International travel ban	Movement restrictions on international travel including air, land, water	0 - No measure 0.33 - Quarantine arrivals from high-risk regions 0.66 - Ban on arrivals from some regions 1- Ban on all regions or total border closure

Some data in this table have been published previously in Knaul, F.M. *et al*. Strengthening health systems to face pandemics: subnational policy responses to COVID-19 in Latin America. *Health Affairs*. **41**, 454–462 (2022)^31^.

Our integrated research teams recorded these data by first reviewing official government websites to capture laws, decrees, and news releases announcing the implementation of each NPI. Each country-team then cross-referenced information from official government sources against news outlets’ coverage of the same laws, decrees, and announcements of NPIs. Finally, our teams performed an additional check of official government social media accounts, such as Twitter and Facebook, when government websites did not announce NPIs. Each week, we performed an internal, random check of intercoder reliability and validity. Two co-authors who were excluded from the original coding independently verified daily data for randomly selected NPI.

We then used the 9 NPI indicators (masks have a separate index) to build a daily composite index score for each national and subnational unit. Creating an index allows for comparison of governments’ overall NPI response to COVID-19 across and within countries.

Our Public Policy Adoption (PPA) Index is constructed by first summing daily scores for each NPI. Then, we account for time by multiplying the sum of NPI scores by a ratio of the days since implementation to the days since a country’s first case. We then weight the measure by estimating the mean PPA score for each administrative unit and weighing it by the population of each state, province, department, or region.

We record data on mask mandates and keep these data separately from the PPA index because the use of face masks behaves differently from the other measures^16^. Mask mandates or recommendations are often a feature of reopening and relaxation of restrictions on population movement. In contrast, mask mandates are designed to moderate the need for physical distancing and allow for closer contact in public and private spaces. Governments often implemented mask mandates and recommendations much later than other NPI, partially based on the WHO’s suggestions for the use of facemasks on June 5, 2020^33^.

### Public policy adoption index

The PPA summarizes governments’ actions and fosters direct comparisons of NPI both within and across countries.

The index is constructed as presented in the following Eq. (1):

1 $${IPP}_{it}=\left\{{\sum }_{j=1}^{n}{I}_{jt}* \left[{\left(\frac{{d}_{ijt}}{{D}_{ijt}}\right)}^{\left(\frac{1}{2}\right)}\right]/10\right\}* 100$$

Whereby:

*IPP*_*it*_ = Public policy adoption index in country/state *i* at time *t*.

*Ij* = Public Policy Index *j*, where *j* goes from 1 to 10.

*D*_*ijt*_ = Days from the first registered case until time *t*.

*d*_*ijt*_ = Days from the implementation of policy *j* until time *t*.

The *IPP*_*it*_ is constructed with the sum of each of the scores from 9 of the 10 NPI, excluding mask mandates, weighted by the day of implementation relative to the first case in each country. The index gives higher scores for earlier implementation relative to the first case in the country. As such, index values rise the earlier an NPI was implemented and the longer it remains in effect.

The ratio *d*_*ijt*_ /*D*_*ijt*_ is continuous and ranges from 0, when policy *j* has not yet been implemented by subnational government *i* at time *t*, to 1, for governments that implemented NPI at the same time *t* when the first COVID-19 case appeared in their country. We then raise the ratio *di*_*jt*_ /*D*_*ijt*_ to the power (1/2) to incorporate decreasing policy effectiveness following delays in NPI implementation.

In the aggregate, each subnational and national government *i* receives a daily score between 0 and 10, which reflects the sum of the different policy dimensions, and is then normalized to a scale of 0 to 100. The maximum index value is 100 but obtaining scores of 100 is unrealistic and, moreover, not necessarily desirable because a score of 100 would imply a complete cessation of activity in an administrative unit following the first case in the country.

We are agnostic about the relative impact of each NPI as well as governments’ rationale for their adoption and weight each NPI equally in the policy index. However, we recognize that NPI adoption and impact might not be equal across interventions and their adoption might stem from different sources. We therefore also construct daily index scores, weighted by time, for the use of facemasks and each of the remaining 9 NPI to allow for assessment of individual NPI as independent or dependent variables. Scores revert to zero when governments remove a policy mandate and return to a score between 0 and 100 as policies resume, with the count of days a policy has been in place beginning from the date of renewed implementation. Users of these data can harness them to assess individual NPIs’ impact on health outcomes, explore their determinants, and compare them to one another.

Our coding for the individual variables is based on a desire for intra- and international comparison at the subnational level and is based on different degrees of policy implementation, ranging from 0 to full. In pursuit of coding clarity and to reflect common distinctions in policy implementation, some variables have three possible values, e.g., no policy, recommended policy, and full policy implementation, whereas others have four. These variables include values for partial implementation, with scores possible between “recommended” NPI and full implementation. We recognize here as well that recommending a policy does not necessarily mean that it will be implemented at half the level of a full policy implementation, which our coding implies. Instead, our coding captures extensive subnational variation, where a state recommends a policy, and some municipalities implement it and others do not. The coding therefore reflects our judgment that a recommended policy is likely to be implemented more than no policy at all, but not as thoroughly as a mandated policy, particularly for NPI whose partial implementation is difficult to measure. This choice represents one of several limitations for creating a cross-national, subnational index that is comparable both within and across countries.

These indices and individual NPI scores translate to 60,129 observations across the 10 original indicators and the index. Additionally, we link our original data to subnational and national information on testing, cases, and deaths, for ease of analysis. These data can be linked further still to inform analysis of a wide variety of COVID-19 outcomes.

**Fig. 1:**
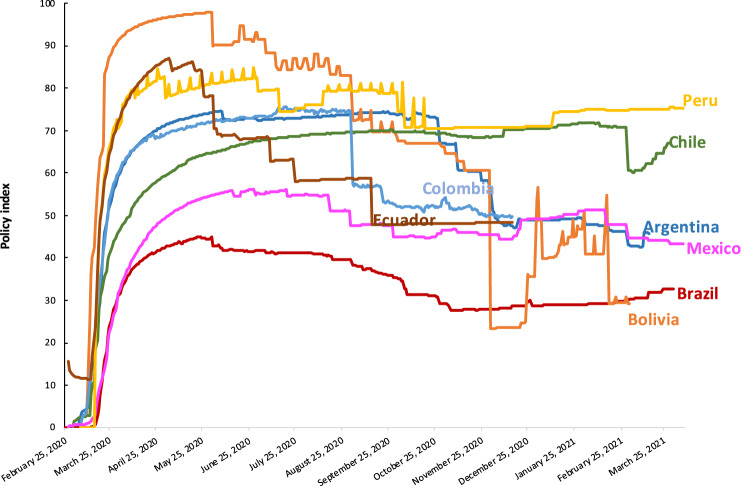
NPI to combat COVID-19 across Latin America. This Figure has been previously published in Knaul, F.M. *et al*. Strengthening health systems to face pandemics: subnational policy responses to COVID-19 in Latin America. *Health Affairs*. **41**, 454–462 (2022)^31^.

**Fig. 2:**
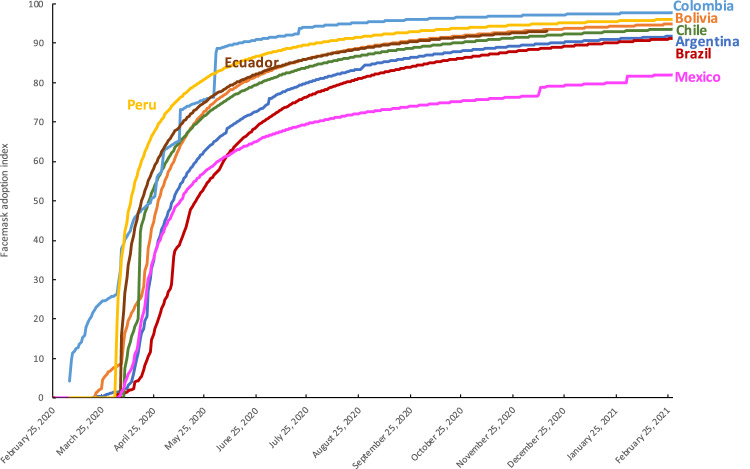
Facemask adoption index across Latin America.

**Fig. 3:**
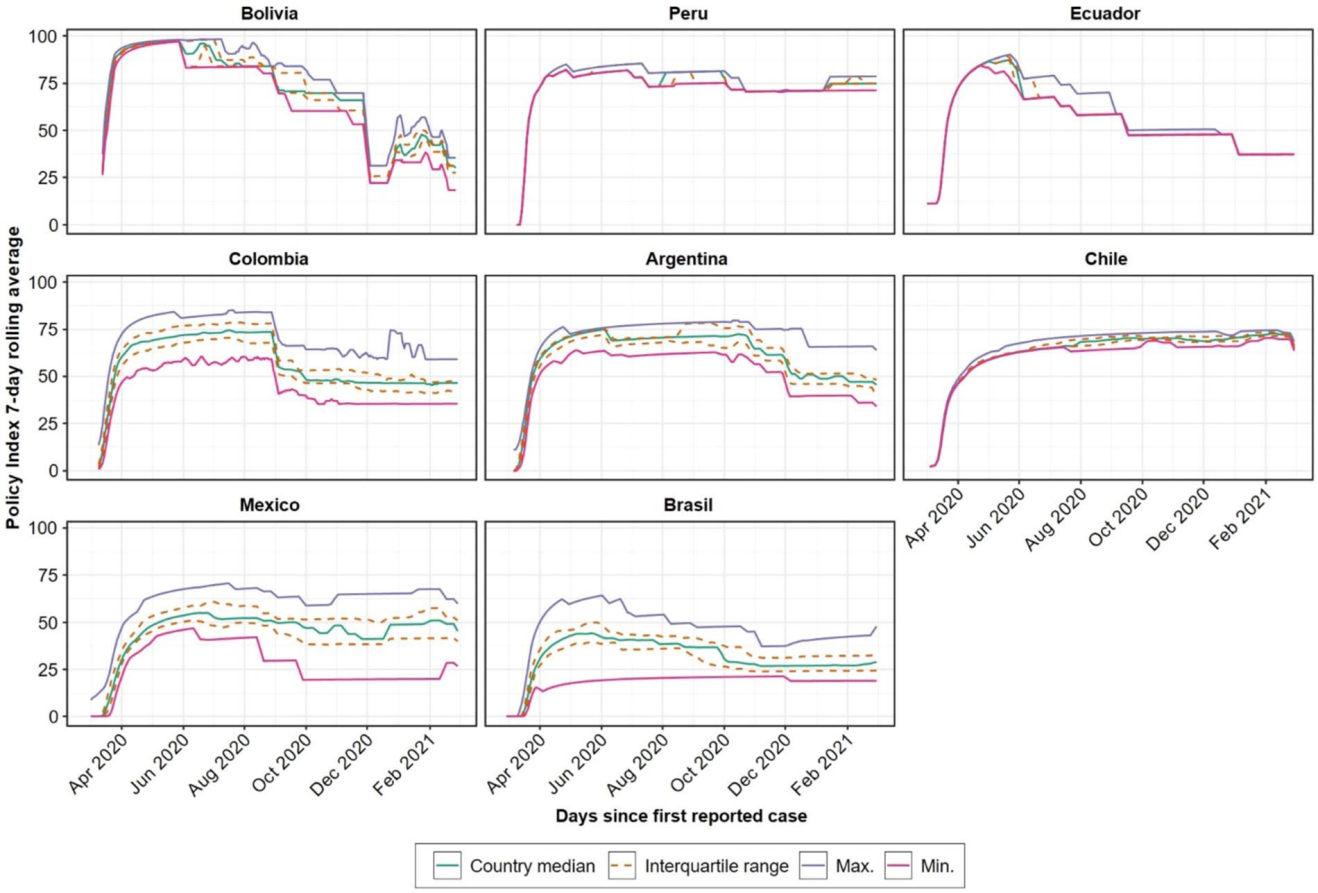
NPI to combat COVID-19 by Individual Country.

Figures 1–3 present different visualizations of the NPI policy index across countries over time, the facemask adoption index, and the policy index by individual country.

## Data Records

Our dataset is available at our Harvard Dataverse under the 10.7910/DVN/NFSXTR The data are in.csv files, divided by country and access is free^16^.

Each row in the dataset corresponds to a subnational government-day, for example, “Brasil, Rio de Janeiro, March 1, 2020.” The country, subnational unit, and date are identified by the column labels “country”, “state_name”, “state_code”, and “date”. “Date is coded as MM/DD/YY across all records. The “days” column counts the days since the first COVID-19 case in each country.

The 10 NPI and the time since their implementation are listed in columns K to AD of the dataset under the variables “School_Closure”, “Days_Since_Schools_Closed”, “Workplace_Closure”, “Days_Workplace_Closure”, “Public_Events_Cancelled”, “Public_Events_Cancelled_Days”, “Public_Transit_Suspended”, “Days_Since_Transit_Suspend”, “Information_Campaign”, “Information_Campaign_Days”, “Internal_Travel_Control”, “Days_Since_Internal_Travel_Ban”, “International_Travel_Controls”, “Days_Since_International_Ban”, “Stay_at_home”, “Days_since_stay_at_home”, “Rest_on_gatherings”, “Days_rest_on_gatherings”, “Use_face_masks”, and “Days_use_face_mask”. The Public Policy Adoption index scores appear in column AF labelled “policy_index”.

## Technical Validation

Each researcher from our integrated country teams coding NPI sent weekly updates to the country-team leaders. These leaders verified sources and coding choices, both for their own countries and in weekly group training sessions as we added country-teams.

Two randomly selected co-authors administered a double-blind review each week during the first four months of data collection and each month thereafter. The two co-authors reviewed randomly selected NPI scores from among each country’s subnational units that members of the country-teams coded. These co-authors then recoded data for a given government on a given day, without having seen the original NPI scores. Neither re-coder knew who coded the original data and no original coder knew which co-author would perform the review. Country teams for which we have data reported discrepancies an average of 6 times per day during the first three months of coding (across 90 daily observations: 10 indicators coded daily across a mean of 9 subnational units). This translates to a 93.7% agreement among double-blind reviewers and a Cohen’s Kappa of 0.75 (high agreement), with growing agreement as coding continued, NPIs stabilized, and were then removed altogether. Note that not all country teams were consistent in the timing or reporting of these data throughout the collection period. We report data from what we argue is a representative sample given the preponderance of data available in each period. Disagreement among coders for all country teams was most common for the “Information Campaign” variable in terms of its partial versus full implementation. Each country-team deliberated in cases of discrepancy, until consensus was reached. Following these checks, country-leaders sent monthly data to the overall project’s data managers.

Next, the project’s data managers checked for missing data, inconsistencies in coding, and mis-entered information by using STATA to perform an automated data assessment. Upon identification, the project’s data managers returned country dataset updates to country-team leaders with embedded queries. Country-teams then updated all scores and return country data to the overall project managers with any inconsistencies or errors resolved.

Project managers then combined country-level data to create a region-wide file that we used to generate monthly country and regional pages that included visualizations of each country’s NPI on each dimension. These materials were posted on the website of the University of Miami Observatory for the Containment of COVID-19 in the Americas, but without the raw data.

We validated the PPA index scores primarily by comparing them to other efforts to track subnational NPI in the Americas during the COVID-19 pandemic. Distinctions in coding methods (research assistants vs. bots, specific indicators for NPI, unit of time (daily vs. weekly or monthly), timeframe (the first year of the pandemic vs. 2020) and construction of our index all suggest that correlations with other indicators will not approach 1. Nevertheless, general assessments of stringency or lack thereof are similar.

We found that our data correlated highly with the Oxford COVID-19 Government Response Tracker^16^. Subnational indicators for NPI were correlated at 0.81 for countries where the Oxford Tracker included subnational data and where indicators, such as the PPA index overlapped with the similar Oxford stringency index. We also compared our index scores with Shvetsova *et al*.’s (2022) Protective Policy Index, which uses automated data collection to generate index scores across all global subnational units, including Latin America, through 2020. The correlation of our index with Shvetsova *et al*.’s is 0.86 for the country-weeks with overlap^28,33^. We provide a table of correlations across indices along with the data and code at the Harvard Dataverse repository^16^.

We used regional and national webinars in May, June, July, and December 2020 as well as February, May, and September 2021, to collect feedback from scholars and practitioners in the region and improve our data coverage. We have also published several peer-reviewed papers using these data, but the full dataset has not been publicly available^1–4,16,32^.

## Usage Notes

We provide replication code in R and in STATA for the ease of the user; the files produce identical calculations. We used the R code for group data collection and updates. We recommend the STATA code for basic replications of our policy index and the R code for evaluating the broader coding effort, the creation of a unified database, and the collaboration across country groups. The R code may also be helpful for other groups engaged in similar research.

## Data Availability

The code used to replicate our index calculations and the creation of all graphics is available at the Harvard Dataverse: 10.7910/DVN/NFSXTR^16^. We provide replication code in R and in STATA for the ease of the user; the files produce identical calculations. We used the R code for group data collection and updates.
