# Author Correction: Non-pharmaceutical interventions to combat COVID-19 in the Americas described through daily sub-national data

**DOI:** 10.1038/s41597-023-02670-6

**Published:** 2023-10-31

**Authors:** Michael Touchton, Felicia Marie Knaul, Héctor Arreola-Ornelas, Thalia Porteny, Óscar Méndez Carniado, Marco Faganello, Calla Hummel, Silvia Otero, Jorge Insua, Fausto Patino, Eduardo Undurraga, Pedro Pérez-Cruz, Mariano Sanchez-Talanquer, V. Ximena Velasco Guachalla, Jami Nelson-Nuñez, Carew Boulding, Renzo Calderon-Anyosa, Patricia J. Garcia, Valentina Vargas Enciso

**Affiliations:** 1 Department of Political Science, School of Arts and Sciences, University of Miami, Miami, Florida USA; 2 Institute for Advanced Study of the Americas, University of Miami, Miami, Florida USA; 3 Leonard M. Miller School of Medicine, University of Miami, Miami, Florida USA; 4 Tómatelo a Pecho, A.C., Mexico City, Mexico; 5 Fundación Mexicana para la Salud, A.C., Mexico City, Mexico; 6 Institute for Obesity Research, Tecnológico de Monterrey, Monterrey, Mexico; 7 School of Government and Public Transformation, Tecnológico de Monterrey, Mexico City, Mexico; 8 Department of Health Policy and Management, Columbia Mailman School of Public Health, New York, New York USA; 9 MAF dataScience, Universidade Estadual de Campinas, Campinas, Brazil; 10 Facultad de Estudios Internacionales, Políticos y Urbanos, Universidad del Rosario, Bogotá, Colombia; 11 Health Policy and Management, School of Biomedical Sciences, School of Government, School of Health Care Management, Universidad Austral, Buenos Aires, Argentina; 12 School of Public Health, University of Buenos Aires, Buenos Aires, Argentina; 13 Universidad Andina, Simón Bolívar, Quito, Ecuador; 14 Escuela de Gobierno, Pontificia Universidad Católica de Chile, Santiago, Chile; 15 Departamento Medicina Interna, Facultad de Medicina, Pontificia Universidad Católica de Chile, Santiago, Chile; 16 Millennium Nucleus for the Study of the Life Course and Vulnerability, Santiago, Chile; 17 Department of Political Science, Colegio de Mexico, Mexico City, Mexico; 18 Department of Government, University of Essex, Essex, England; 19 Department of Political Science, University of New Mexico, Albuquerque, New Mexico USA; 20 Department of Political Science, University of Colorado, Boulder, Colorado USA; 21 Department of Epidemiology, Biostatistics and Occupational Health, McGill University, Montreal, Quebec Canada; 22 Universidad Peruana Cayetano Heredia, Lima, San Martin de Porres Peru

**Keywords:** Research data, Interdisciplinary studies, Policy, Infectious diseases, Public health

Correction to: *Scientific Data* 10.1038/s41597-023-02638-6, published online 21 October 2023

In this article the funding from ‘the University of Miami Institute for Advanced Study of the Americas and Tecnologico de Monterrey (Challenge-Based Research Funding Program, I036-IOR005- C5-T3-T)’ was omitted. The original article has been corrected.

Additionally, the affiliation ‘Institute for Obesity Research, Tecnológico de Monterrey, Monterrey, Mexico’ was incorrectly assigned for Felicia Marie Knaul. The original article has been corrected.

